# Physical aggression toward parents from ages 11 to 24: prevalence trajectory and risk and protective factors

**DOI:** 10.1007/s00787-025-02953-w

**Published:** 2026-01-19

**Authors:** Laura Bechtiger, David Bürgin, Gregor Ferolla Vasconcelos, Denis Ribeaud, Manuel Eisner, Lilly Shanahan

**Affiliations:** 1 Jacobs Center for Productive Youth Development, University of Zurich, Andreasstrasse 15, Zurich, 8050 Switzerland; 2 Child and Adolescent Psychiatric Research Department, University Psychiatric Hospitals, University of Basel, Basel, Switzerland; 3 Institute of Criminology, University of Cambridge, Cambridge, UK; 4 Department of Psychology, University of Zurich, Zurich, Switzerland

**Keywords:** Family aggression, Youth-to-parent aggression, Prevalence, Longitudinal cohort, Risk and protective factors, Adolescence

## Abstract

Physical youth-to-parent aggression (PYPA) is among the most understudied forms of family aggression in the general population. This study examined the prevalence and developmental course of PYPA from adolescence into young adulthood and investigated childhood behavioral and interpersonal risk (e.g., ADHD symptoms, low self-control, harsh parenting) as well as protective factors (e.g., conflict coping, parental involvement). Data came from six assessments of the prospective-longitudinal Zurich Project on Social Development from Childhood to Adulthood (z-proso), including *N* = 1522 participants. Participants reported PYPA at ages 11, 13, 15, 17, 20, and 24. Risk and protective factors were measured at or before age 11. Generalized estimating equations were used to examine the developmental course of PYPA, and logistic regression analyses examined its risk and protective factors. One in three youth (32.5%) reported engaging in PYPA at least once. Prevalence followed a cubic trajectory: increasing from age 11 to 13, then declining at varying rates through age 24. ADHD symptoms (OR (95%CI) = 1.26 (1.10, 1.45)), harsh parenting (OR (95%CI) = 1.24 (1.08, 1.42)), and interparental disagreement (OR (95%CI) = 1.17 (1.01, 1.35)) were associated with higher odds of PYPA; whereas competent conflict coping (OR (95%CI) = 0.83 (0.72, 0.96)) and parental involvement (OR (95%CI) = 0.83 (0.72, 0.95)) were associated with lower odds after accounting for participants’ aggressive tendencies during childhood. Reducing children’s aggressive tendencies and exposure to aggression and conflict, while fostering competent coping skills and supportive family environments could decrease PYPA and the burden of family aggression.

**Supplementary Information:**

The online version contains supplementary material available at 10.1007/s00787-025-02953-w.

Parental aggression (e.g., corporal punishment) and witnessing inter-parental aggression are linked to adverse outcomes among children that can persist into adulthood and across generations [1–4]. Physical aggression in families is not limited to aggression initiated by parents. Physical youth-to-parent aggression (e.g., hitting or throwing things; PYPA) is commonly reported in clinical, child-welfare, and juvenile justice settings, with a prevalence rate of up to 85% [5–7]. In the general population, prevalence estimates range from 5 to 21% and are mostly derived from cross-sectional studies of adolescents (9 to 17 years) [5]. How the prevalence of PYPA changes across adolescence and early adulthood is not understood, but change would be expected given developmental shifts in aggression and family dynamics during these periods: In contrast to other antisocial behaviors (e.g., delinquency and conduct disorder), children’s physical aggression declines from early childhood into adolescence [8–10]. At the same time, parent-child relationships are renegotiated during adolescence, with temporary increases in conflict in some families [11–13]. The only longitudinal examination of PYPA suggests an increase in PYPA from ages 13 to 15, followed by a decrease until age 17 [14]. This study did not extend into adulthood. Accordingly, our first aim was to examine age-related changes in the prevalence of PYPA from early adolescence to early adulthood in the general population.

## Risk and protective factors for physical youth-to-parent aggression

To develop effective preventions aimed at reducing family aggression, a better understanding of childhood risk and protective factors of PYPA is needed. Existing research relies on cross-sectional designs and, therefore, is not informative regarding temporal ordering.

### Risk factors

Cross-sectional research on the sociodemographic correlates of PYPA has provided mixed results. Some studies suggest that PYPA is more prevalent in families with higher socioeconomic status (SES), while others report no such association [5, 15–17]. Furthermore, some studies have found PYPA to be more prevalent among families from majority backgrounds, such as European American families in the United States and native German families in Germany, compared to families from racial/ethnic minority or migrant backgrounds [5, 17]. This could reflect different cultural norms regarding parent-child relationships or more socially desirable reporting among minoritized groups. Moreover, some studies suggest that single parenthood is associated with increased PYPA, perhaps reflecting the availability of fewer psychosocial resources when families can rely on only one parent rather than two [5].

PYPA also occurs in contexts of **behavioral risk** [5]. This includes aggressive tendencies (e.g., toward other people in general or following provocation), engaging in delinquent behaviors and early substance use, and could also extend to low self-control, which is a common risk factor for antisocial behavior [14, 16–20]. Furthermore, children with elevated symptoms of attention-deficit hyperactivity disorder (ADHD) show more aggressive behaviors as compared to other children, likely because of difficulties with impulse control [21]. Indeed, in adolescents in child-welfare or juvenile justice instutions, ADHD is commonly diagnosed in youth who engage in PYPA [22, 23].

Third, research on the cycle of violence indicates that children’s and adolescents’ **exposure to aggression and conflict** predicts future aggressive behavior through mechanisms such as social learning, altered attachment, and neurobiological and (epi-)genetic changes [4]. Exposure to harsh parenting (e.g., corporal punishment and verbal aggression) and witnessing disagreement and conflict between parents has consistently been linked with increased aggression and PYPA [5, 15, 17, 24]. Exposure to aggression and conflict outside the family has not previously been considered in relation to PYPA. However, bullying victimization, serious assault victimization, and exposure to aggression and conflict at school are common experiences in early adolescence that may be linked to PYPA [25].

### Protective factors

To date, the factors that shield families from PYPA have not been examined. **Behavioral and psychological protective factors** likely include effective coping strategies and emotional well-being. Individuals who cope with negative emotions and conflict in competent and non-aggressive ways are less likely to become frustrated and act out as compared to others [26, 27]. Similarly, more emotional well-being (i.e., having fewer symptoms of anxiety and depression) is associated with better emotion regulation skills and could therefore reduce behavioral and family dynamics that could contribute to PYPA [28]. In the interpersonal domain, supportive relationships with parents, teachers, and peers are important **relationship resources** for behavioral regulation, as interactions with others provide opportunities for young people to learn effective strategies for coping with interpersonal conflict [28, 29]. Our second aim was to extend prior literature by considering a comprehensive set of multi-domain childhood risk and protective factors for PYPA in a prospective-longitudinal community sample.

### Additional considerations: general aggression and sex differences

Many of the risk factors documented for PYPA overlap with those for aggressive tendencies in general [5, 30, 31]. However, with a few exceptions [16, 18], previous investigations of PYPA have not accounted for these factors. Whether general aggressiveness and PYPA reflect a common latent trait or represent separate but closely related phenomena, the question remains whether the identified risk factors indicate a general vulnerability for interpersonal aggression, regardless of the victim, or are specific to PYPA.

Physical aggression perpetration in youth is typically more prevalent in males than in females [30, 32]. Meanwhile, sex differences in PYPA in the general community are comparatively small [5]. Whether this lack of sex differences in the prevalence of PYPA extends to its developmental course and etiology is unclear. Our final aim was to consider general aggression and examine sex differences in the prevalence of PYPA, as well as its risk and protective factors for PYPA.

## Methods

### Sample description

Data came from the prospective-longitudinal cohort study *Zurich Project on Social Development from Childhood to Adulthood (z-proso)*. A detailed study description is available elsewhere [33]. In 2004, z-proso sampled 1,675 first graders from schools in the city of Zurich using a cluster-randomized sampling approach. Characteristic of the population of Zurich, two thirds of focal participants’ parents were born abroad (in > 80 countries), although ~ 90% of focal participants were born in Switzerland. z-proso began as a cluster RCT to evaluate two preventive interventions aimed at reducing aggression and delinquency. The intervention effects were largely non-significant [34, 35], but see [36] for educational outcomes. All intervention groups have been followed up repeatedly at ages 7, 8, 9, 11, 13, 15, 17, 20, and, at the latest assessment in 2022, at age 24. At ages 20 and 24, 85.4% and 47.3% of participants reported living with their parents, respectively.

Participants’ parents and main teachers contributed data until age 11 and age 17, respectively. From age 11 to 17, focal participants completed paper-and-pencil questionnaires in schools. At ages 20 and 24, participants completed computer-administered surveys in a university research laboratory (*n* = 1143 and *n* = 987, respectively) or participated online (*n* = 37 and *n* = 173, respectively). Participants received monetary compensation, which increased from ~ 35USD at age 13 to ~ 175USD at age 24, when many participants were part of the workforce. At age 11, parents received a shopping voucher worth 50CHF. Since 2017, z-proso receives approval from the Ethics Committee of the Faculty of Arts and Social Sciences at the University of Zurich. Before 2017, ethics approval for the study was not necessary under Swiss law, but all study procedures were compliant with national and institutional ethical standards and the Helsinki Declaration of 1975 as revised in 2008.

### Measures

***PYPA*** was self-reported by youth at ages 11, 13, 15, 17, 20, and 24 with two items created by the z-proso team: “How often did you hit or kick your parents in anger?” and “How often did you throw things at your parents in anger?” Participants reported the frequency of these behaviors in the past 12 months from 1=“never” to 5=“very often.” Responses to both items were highly skewed; therefore, binary prevalence variables were created to indicate whether participants endorsed either item, coded 1=“at least rarely” vs. 0=“never” at each age. For the regression analyses, the cumulative prevalence of PYPA was calculated that indicated whether participants reported PYPA at least once across ages 13 to 24 (coded as 1) versus never (coded as 0).

Detailed measures descriptions of ***childhood risk and protective factors*** are presented in the eTable 1. Unless otherwise specified, risk and protective factors were assessed at age 11. All mean scores were z-standardized for better comparability. **Demographic risk factors** included child sex assigned at birth, family SES (reported at ages 11, 13, and/or 15), migration background (reported at ages 11, 13, and/or 15), and parental separation/divorce. **Behavioral risk factors** included ADHD symptoms, low self-control, engaging in delinquent behaviors, any substance use, and bullying perpetration. Children’s general aggression was also measured at age 11. **Interpersonal risk factors** related to experiencing aggression and conflict within the home (i.e., harsh parenting, poor parental relationship quality, and parental disagreement), with peers (i.e., bullying victimization, school-level problems), and experiencing serious victimization. Poor parental relationship quality and parental disagreement were parent-reported at child age 8.

**Behavioral protective factors** included stress coping abilities (i.e., low aggressive conflict coping and competent conflict coping) and emotional well-being. **Interpersonal protective factors** included relationship resources such as parental involvement, teacher bonding, and class bonding.

Assigned intervention group was included as a covariate in regression analyses with the control group as the reference group.

### Data analysis

Prevalence estimates for PYPA were calculated for the overall sample and by sex. To examine the shape of the prevalence trajectory across age, generalized estimating equations (GEEs) were specified in SPSS version 28.0.1.1. Linear (age), quadratic (age^2^), and cubic (age^3^) trends were examined. Age was z-standardized before computing polynomial terms to increase scale comparability. GEEs account for the non-independence of repeated observations by using robust standard-errors. The cubic time trend was the highest-order trend significantly associated with PYPA; therefore we present these results. To evaluate sex differences, age*sex, age^2^*sex, and age^3^*sex interactions were specified.

Logistic regression models were estimated separately for each childhood risk and protective factor predicting the cumulative prevalence of PYPA between ages 13 and 24, adjusted for intervention group, sex, family SES, and migration background (Model 1). Risk/protective factors*sex interactions were added to the regression models to examine sex differences. Next, regression analyses were re-run, also adjusting for children’s general aggression at age 11 (Model 2). All regressions were estimated in Mplus 8.10 using maximum likelihood estimation with robust standard errors (MLR).

### Attrition and missing data

Overall, *n* = 1,147 participated at the age 11 assessment, *n* = 1,365 at age 13, *n* = 1,446 at age 15, *n* = 1,305 at age 17, *n* = 1,180 at age 20, and *n* = 1,160 at age 24. This corresponds to 68.5%, 81.5%, 86.3%, 77.9%, 70.4%, and 69.3% of the original target sample (*N* = 1,675) and 72.5%, 86.2%, 91.4%, 82.4%, 74.5%, and 73.3% of the *n* = 1,583 who contributed data at least once via at least one informant, respectively. Participation was highest at age 15 because all participants could be recontacted and active parental consent was no longer required after age 13. Attrition over time was associated with participant characteristics. Males, youth whose parents were both born outside Switzerland, and those from families with a lower SES were more likely to drop out of the study [33, 37]. Among participants who provided at least one valid PYPA value (*N* = 1,522), we examined whether those who reported PYPA at every assessment from age 11 to 24 (*n* = 735) differed on age 11 study variables from those with at least one missing PYPA value (*n* = 787). Those with complete case data reported lower ADHD symptoms, more competent conflict coping, higher levels of parental disagreement, more bullying victimization and lower class bonding than those with partial missing data (see eTable 2 for an overview). For a more dynamic examination of attrition in z-proso across the different survey waves, see Eisner et al. [37]. A summary of missing data is also provided in the eSupplement (eTable 3).

For prevalence and GEEs, all available data at any given assessment were used. For the regression analyses, 75 data sets were imputed using Bayesian estimation stratified by sex for all participants who provided at least one valid value of PYPA (*N* = 1,522; see also eSupplement and eTable 4 for more information about the imputation model). The reported coefficients were pooled according to Rubin’s rules. For all analyses, two-sided tests of statistical significance were performed at an α-level of 0.05 (95% confidence intervals). We report complete case analysis as a sensitivity analysis.

## Results

**Fig. 1:**
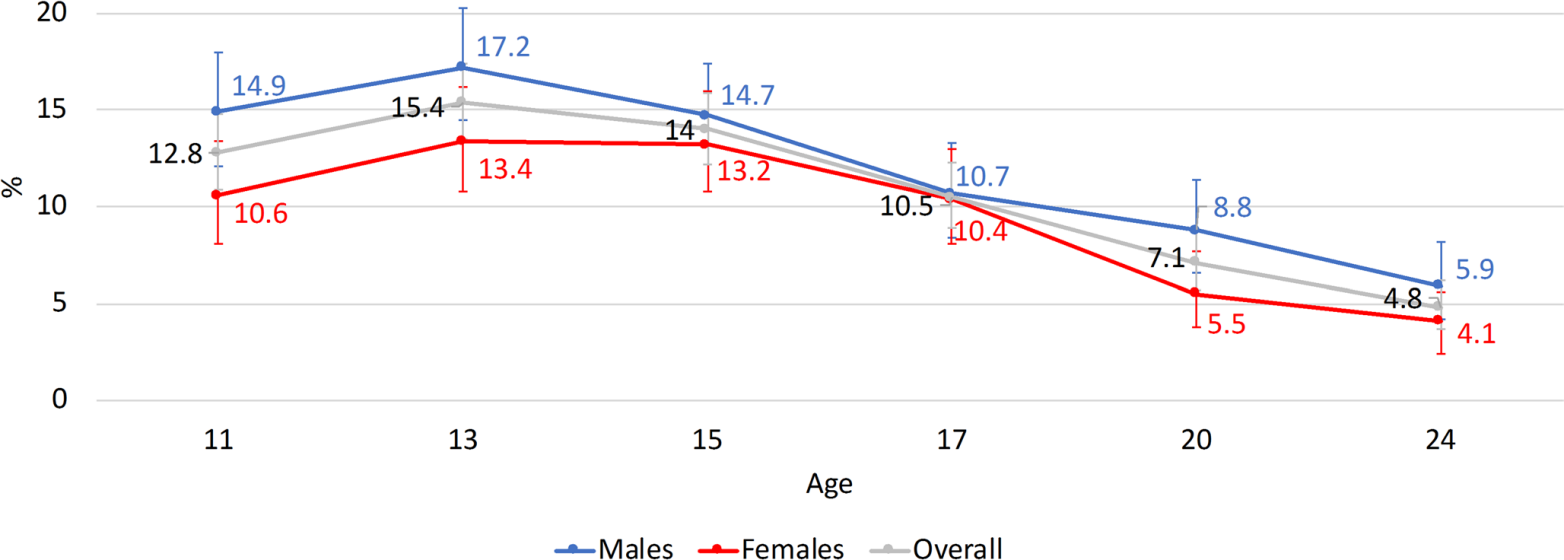
Prevalence of youth-to-parent aggression from age 11 to age 24 for the overall sample, males, and females. N ranges from 1136 to 1438 for the overall sample, from 572 to 743 for males and from 558 to 695 for females

Descriptive statistics of study variables are presented in eTable 5. The cumulative prevalence of PYPA between ages 11 and 24 across all assessments was n/*N* = 495/1522 (32.5%). Of those who engaged in PYPA at least once, *n* = 297 (60%) reported it at one assessment, *n* = 109 (22%) at two assessments, and *n* = 89 (18%) at three or more assessments. Prevalence rates peaked at age 13 (see Fig. 1 and eTable 6), when n/*N* = 208/1355 (15.4%) engaged in PYPA. Subsequently, the prevalence decreased and flattened in young adulthood. At age 24, n/*N* = 56/1159 (4.8%) still engaged in PYPA. GEEs confirmed this cubic trajectory (OR age = 0.54, 95%CI = 0.45–0.65; OR age^2^ = 0.81, 95%CI = 0.74–0.88; OR age^3^ = 1.17, 95%CI = 1.07–1.28). Although the individual polynomial age coefficients from the GEE are not directly interpretable in isolation, collectively they indicate an initial increase in prevalence during early adolescence, followed by a decline across late adolescence and a leveling off in young adulthood. Those who reported PYPA at one assessment were much more likely to report PYPA at the next assessment, with ORs ranging from 6.86, 95%CI = 4.57–10.29, to 17.86, 95%CI = 9.61–33.19 (Table 1).

**Table 1:** Stability of PYPA from one assessment to the next

		95%CI	
	OR	Lower	Upper
PYPA 11→ PYPA 13	**6.86**	4.57	10.29
PYPA 13→ PYPA 15	**6.61**	4.72	9.25
PYPA 15→ PYPA 17	**13.11**	8.90	19.32
PYPA 17→ PYPA 20	**10.43**	6.49	16.77
PYPA 20→ PYPA 24	**17.86**	9.61	33.19

*N* = 1522. The increasingly large confidence intervals are likely attributable to small cell sizes of participants who report PYPA at both time points

The cumulative prevalence of PYPA between ages 11 and 24 was n/*N* = 279/784 (35.6%) for males and n/*N* = 216/738 (29.3%) for females, respectively (OR = 1.34, 95%CI = 1.08–1.66). Figure 1 shows that males were more likely to engage in PYPA than females at ages 11 (OR = 1.48, 95%CI = 1.04–2.11), 13 (OR = 1.35, 95%CI = 1.00–1.82), and 20 (OR = 1.65, 95%CI = 1.05–2.60) with a significant cubic time course for both sexes (OR age = 0.58, 95%CI = 0.46–0.75 and 0.49, 95%CI = 0.37–0.66; OR age^2^ = 0.87, 95%CI = 0.78–0.97 and 0.73, 95%CI = 0.64–0.84; OR age^3^ = 1.12, 95%CI = 1.002–1.26 and 1.22, 95%CI = 1.06–1.40, for males and females respectively). None of the sex*age interactions were significant.

### Risk and protective factors

Table 2 presents results of the logistic regression analyses (Model 1). None of the sociodemographic characteristics were significantly associated with increased odds of PYPA. In the behavioral risk domain, more ADHD symptoms, child general aggression, lower self-control, more delinquency, any substance use, and PYPA at age 11 were significantly associated with cumulative PYPA from 13 to 24. Bullying perpetration was not significantly associated with subsequent PYPA. Among interpersonal risk factors, exposure to harsh parenting and serious victimization at age 11, and poor interparental relationship quality and greater parental disagreement at age 8 were associated with PYPA. This was not the case for bullying victimization and school problems.

**Table 2:** Odds ratios from logistic regression models of risk factors at age 11 predicting cumulative PYPA (age 13–24)

Risk factors	Model 1				Model 2		
	OR	L95%CI	U95%CI	Sex interaction (p)	OR	L95%CI	U95%CI
**Control variables and sociodemographic risk factors**							
Intervention: PATHS	0.78	0.57	1.06	--	**0.72**	0.53	0.99
Intervention: PATHS & Triple P	0.73	0.52	1.01	--	**0.71**	0.50	0.99
Intervention: Triple P	**0.59**	0.43	0.82	--	**0.57**	0.40	0.80
Male sex	1.25	0.99	1.57	--	1.00	0.78	1.29
SES	0.95	0.84	1.09	0.477	0.96	0.84	1.10
Migration background	1.07	0.83	1.38	0.544	1.06	0.81	1.37
Parental separation by age 11	1.10	0.81	1.50	0.441	1.03	0.75	1.41
**Behavioral risk factors**							
ADHD symptoms	**1.30**	1.13	1.49	0.752	**1.25**	1.09	1.44
General aggression	**1.50**	1.31	1.72	0.319	--	--	--
Low self-control	**1.24**	1.08	1.42	0.271	1.00	0.85	1.18
Delinquency	**1.19**	1.05	1.35	0.787	1.02	0.89	1.17
Any substance use	**1.65**	1.06	2.58	0.190	1.31	0.81	2.12
Bullying perpetration	1.08	0.95	1.24	0.571	**0.84**	0.72	0.99
PYPA 11	**4.91**	3.35	7.20	0.151	**4.14**	2.79	6.16
**Aggression and conflict exposure**							
Harsh parenting	**1.37**	1.21	1.56	0.295	**1.23**	1.07	1.41
Poor parental relationship quality^a^	**1.17**	1.01	1.36	0.082	1.12	0.96	1.31
Parental disagreement^b^	**1.21**	1.05	1.38	0.143	**1.17**	1.01	1.35
Bullying victimization	1.12	0.99	1.28	0.749	1.01	0.88	1.16
Serious victimization	**1.27**	1.12	1.44	0.574	**1.17**	1.02	1.34
School problems	1.11	0.97	1.26	0.940	1.08	0.95	1.23
**Stress coping and emotional well-being**							
Competent conflict coping	**0.79**	0.70	0.90	0.515	**0.85**	0.74	0.98
Low aggressive conflict coping	**0.83**	0.73	0.95	0.602	1.13	0.95	1.34
Emotional well-being	0.91	0.79	1.04	0.407	1.03	0.89	1.19
Relationship resources							
Parental involvement	**0.80**	0.70	0.92	0.195	**0.84**	0.73	0.96
Teacher bonding	**0.87**	0.76	0.99	0.682	0.98	0.85	1.13
Class bonding	**0.87**	0.77	0.99	0.130	0.95	0.83	1.09

SES = socioeconomic status. ADHD = attention-deficit hyperactivity disorder. PYPA = physical youth-to-parent aggression. Model 1 regressions are adjusted for intervention group, sex, SES, and migration background. The p-value for the risk/protective factor*sex interaction refers to the p-value of the interaction coefficient specified in additional models. Model 2 regressions are additionally adjusted for general aggression at age 11. The reported coefficients for intervention groups, sex, SES, and migration background come from the same basic model. Significant associations (at *p* < .05) are bolded. Missing data was handled with multiple imputation stratified by sex (m = 75 data sets). Poor parental relationship quality and parental disagreement were not imputed as not to impute data for parents not living with a partner or co-parenting, respectively

^a^ *N* = 951 because not all parents were living with a partner

^b^ *N* = 1014 because not all parents were living with a partner or co-parenting

For behavioral protective factors, competent and low aggressive conflict coping, but not emotional well-being, were associated with a lower likelihood of PYPA. For relationship resources, parental involvement, teacher bonding, and class bonding were associated with a lower likelihood of PYPA. None of the risk/protective factors*sex interaction terms were significant. With the exception of PYPA, which was associated with increased odds for later PYPA by more than four, most associations were small to moderate in size, with significant odds ratios ranging from 1.17 to 1.65 for increased risk, and from 0.79 to 0.87 for decreased risk (see Table 2 for all coefficients).

After adjusting for general aggression (Model 2) to identify risk factors specific to PYPA rather than aggression overall, only a subset of risk and protective factors remained significant (Table 2). For behavioral risk factors, ADHD symptoms and PYPA at age 11 remained significant. For bullying perpetration, a significant negative association emerged. Among interpersonal risks, harsh parenting, parental disagreement, and serious victimization remained significant. Competent conflict coping was the only behavioral protective factor, and parental involvement the only relationship resource that remained negatively associated with PYPA. Most associations were small to moderate in magnitude (see Table 2 for odds ratios).

These results were mostly replicated in follow-up analyses that additionally controlled for PYPA at age 11, suggesting that the examined risk and protective factors predicted an onset of PYPA after that age (eTables 7 and 8). To examine whether protective factors reduce the likelihood of PYPA for all participants or specifically for those already engaged at age 11, we included interaction terms between each protective factor and PYPA at age 11. Interactions were nonsignificant, suggesting that these factors decrease the likelihood of PYPA for everyone. We report complete case analysis (*n* = 735) of the main models in the eSupplement (eTable 9). Results were mostly replicated.

## Discussion

This study examined the prevalence of and childhood risk and protective factors for PYPA during adolescence and young adulthood in a prospective-longitudinal community cohort. The results revealed that PYPA affects many families in the general population, with about one-third of young people engaging in PYPA at least once between the ages of 13 and 24. This is likely an underestimate given that our six assessments asked the respondents about PYPA only in the past year. Furthermore, PYPA was assessed using only two items (i.e., hitting or kicking and throwing things); thus, the entire spectrum of PYPA behaviors was not addressed.

Our study is among the first to examine the developmental course of PYPA, identifying a cubic course characterized by an initial increase followed by a subsequent decrease at varying rates. This is consistent with the developmental trajectory of other antisocial behaviors and dual-system theories that highlight early adolescence as a period of heightened impulsivity [30, 38, 39]. The stability of PYPA from one assessment to the next was highest from ages 20 to 24 years, suggesting that young adults who engage in PYPA are at a high risk of continuing this behavior over time. This finding is consistent with Moffitt’s taxonomy of antisocial behavior, which suggests that while antisocial behavior is common during adolescence, only a minority of adolescents continue this behavior into young adulthood [10]. Even this small percentage of young adult PYPA is concerning given the physical strength of young adults.

We identified behavioral and interpersonal risk factors for PYPA. The former included ADHD symptoms, general aggression, low levels of self-control, delinquent behavior, and early substance use; the latter included interpersonal risk, harsh parenting, poor parental relationships, parental disagreement, bullying victimization, and serious victimization. These risk factors remained significant after controlling for PYPA at age 11, suggesting that they are also associated with the onset of PYPA. Associations with sociodemographic variables were weak. PYPA thus affects all segments of the population equally [5].

Most associations between risk and protective factors and PYPA were explained by general aggression, which could act as a mediator [30, 40]. Nevertheless, ADHD symptoms, experiencing harsh parenting and parental disagreement, and being a victim of a serious assault remained uniquely associated with PYPA regardless of individual differences in general aggression and thus had specific associations with PYPA. This confirms existing research on the cycle of violence that documents the detrimental impact of harsh parenting and other forms of violence on child development [1, 3]. Furthermore, ADHD symptoms, which are linked to reduced behavioral regulation, and victimization experiences outside of the family, must be considered as potential points for prevention and intervention regarding family aggression. After we adjusted for general aggression, a negative association emerged between bullying perpetration and a lower likelihood of PYPA. This suggests that children may be less likely to engage in aggression against parents when they aggress against peers. However, we must interpret this association with caution, as it only appears after adjusting for general aggression, suggesting complex relationships among the covariates.

This study is among the first to examine protective factors in the context of PYPA. Less aggressive conflict coping and positive relationships with parents, teachers, and classmates were initially associated with a lower risk of PYPA. Competent conflict coping and parental involvement were still associated with low PYPA after adjusting for general aggression. Thus, when conflict situations emerge in families, both parental and personal resources may be needed to decrease the risk of PYPA. Follow-up analyses suggested that these factors reduce the likelihood of PYPA for everyone, rather than reducing the likelihood of re-engaging in PYPA. PYPA could signal that youth are unable to adequately cope with their emotions (i.e., anger) and lack supportive relationships.

Although we identified some sex differences regarding PYPA (i.e., a higher cumulative prevalence and higher prevalences at ages 11, 13, and 20 for males), these are smaller than those for physical aggression in general, which is consistent with previous PYPA research in community samples [5, 32, 41]. We were unable to consider the severity PYPA; however, previous research shows that males perpetrate more severe PYPA than females [23, 42]. The associations with risk and protective factors did not differ by sex, suggesting shared etiologies of PYPA for males and females.

### Strengths and limitations

The strengths of this study include the six repeated measures of PYPA across multiple developmental periods in a population-based sample, allowing us to examine a comprehensive set of risk and protective factors. The limitations of this study include, first, its focus on *physical* aggression, without considering other forms of youth-to-parent aggression (e.g., verbal and emotional aggression) [43]. Also, our measure of PYPA includes only two items and captures acts of aggression perpetrated “in anger.” Thus, it primarily captures aggression that emerges from situations that involve escalating conflict. Although this measure of PYPA is not ideal, to our knowledge, this is the only study to include repeated measures of PYPA across a 13-year timespan from early adolescence to young adulthood. Second, data on PYPA before age 11 were unavailable, and we could not document its prevalence during childhood. Third, PYPA was self-reported. While this could have led to underreporting because of embarrassment or social desirability, parental reports would have likely led to the underreporting of PYPA due to feelings of shame and being held responsible for their child’s behavior [44]. Fourth, we examined risk factors for PYPA at a single point in time, rather than focusing on developmental processes. The processes leading to PYPA in situations of anger likely also include short-term and situational processes. Future research should combine such predictors across various timeframes to understand the interplay between short- and long-term influences on family aggression.

### Conclusion and implications

Many families are affected by PYPA during adolescence and young adulthood, most often in early to mid-adolescence. PYPA occurs in a context characterized by behavioral risk, aggression and conflict exposure across contexts, and a lack of coping skills and relationship resources. Some of these associations are accounted for by children’s general aggression. Reducing children’s aggressive tendencies, exposure to aggression, promoting competent coping abilities, and creating supportive family environments could lower the burden of family aggression. Considering the increasing stability of PYPA toward later adolescence and young adulthood, prevention and identification efforts must begin early.

## Supplementary Information

Below is the link to the electronic supplementary material.

Supplementary Material 1 (DOCX 96.5 KB)

## Data Availability

From Fall 2024, z-proso data is successively available through SWISSUbase and can be requested by researchers through this platform (link to the project: https://www.swissubase.ch/de/catalogue/studies/9707/20382/overview). All users are required to sign a confidentiality agreement with the University of Zurich or a user agreement with SWISSUbase to access the data. Researchers can also request the data not yet made available on SWISSUbase from the principal investigators of the z-proso project with a brief sketch of its planned use.
