# Supplementary material for: Physical aggression toward parents from ages 11 to 24: prevalence trajectory and risk and protective factors
Source: Eur Child Adolesc Psychiatry. 2026 Jan 19;35(5):1545–53. doi: 10.1007/s00787-025-02953-w (PMC13272229; doi:10.1007/s00787-025-02953-w)
Supplement: Supplementary file 1 — Supplementary Material 1 (DOCX 96.5 KB) [file 787_2025_2953_MOESM1_ESM.docx]

**Supplementary Material: Physical Aggression toward Parents from Ages 13 to 24: Prevalence Trajectory and Risk and Protective Factors**

*Laura Bechtiger^1^,PhD, David Bürgin^1,2^, PhD, Gregor Ferolla Vasconcelos^1^, M.Sc., Denis Ribeaud^1^, PhD, Manuel Eisner^1,3^, PhD, Lilly Shanahan^1,4^, PhD*

Index

[eTable 1. Measures description of risk and protective factors 2](#_Toc206489874)

[eSupplement 1. Attrition, missing, data, and imputation 8](#_Toc206489875)

[eTable 5. Descriptive statistics for those who ever engaged in PYPA and those who did not engage in PYPA. 12](#_Toc206489876)

[eTable 6. Prevalences of PYPA in the overall sample, and by sex, with confidence intervals 14](#_Toc206489877)

[eTable 7. Sensitivity analysis adjusting for PYPA at age 11, intervention group, and sociodemographics 15](#_Toc206489878)

[eTable 8. Sensitivity analysis adjusting for PYPA at age 11, general aggression, intervention group, and sociodemographics 16](#_Toc206489879)

[References 17](#_Toc206489880)

# **eTable 1. Measures description of risk and protective factors**

| **Construct** | **Measures description** | **Cronbach’s α** | **Scale/Coding** |
| --- | --- | --- | --- |
| **Covariates and demographic risk factors** | | | |
| Assigned intervention group | Consistent with the original cluster-randomized design of z-proso, one of four intervention groups was randomly assigned on a school level (i.e., all students entering first grade in the same school were assigned to the same intervention group). The four intervention groups were PATHS, Triple P, PATHS and Triple P, and a control group. For more information regarding the intervention design and content, see the z-proso cohort profile [1]. Assigned intervention group was included as a covariate in the form of three dummy variables with the control group serving as the reference group. |  | 3 dummy coded variables: PATHS (0= no, 1=yes), PATHS & Triple P (0= no, 1=yes), Triple P (0=no, 1=yes) |
| Child sex | Child sex assigned at birth was available from the official school records. | -- | Binary variable (0=female; 1=male) |
| Family SES | Family SES was assessed using the International Socio-Economic Index of Occupational Status [2] based on parental occupation reported by focal participants. This score captures occupation-specific income and required educational level. A composite score was created based on information from when participants were 11, 13, and 15 to maximize information. | -- | 16 (e.g., unskilled worker) to 90 (e.g., judge) |
| Migration background | Migration background was assessed using parental country of birth reported by focal participants. A composite score was created based on information from when participants were 11, 13, and 15 to maximize information. | -- | Binary variable (0= at least one parent born in Switzerland; 1= both parents born abroad) |
| Parental separation/divorce | Parental separation/divorce was reported by focal participants at age 11. | -- | Binary variable (0= not separated/divorced; 1= separated/divorced) |
| **Behavioral risk factors** | | | |
| ADHD symptoms | ADHD symptoms were parent-reported on 9 items from the Social Behavior Questionnaire [3]. Parents rated their child’s inattentive (e.g., “CHILD can’t concentrate or pay attention for long”) and hyperactive (e.g., “CHILD is impulsive, acts without thinking”) behaviors. | 0.85 | Mean score: five-point Likert scale from 1=“never” to 5=“very often” |
| General aggression | General aggression was self-reported on 9 items from the SBQ [3,4] and comprises the subscales proactive aggression (3 items, e.g. “You intimidated others to get what you want.”), physical aggression (3 items, e.g., “You physically attacked other people”), and reactive aggression (3 items, e.g., “You got very angry when someone teased or irritated you”). | 0.77 | Mean score: five-point Likert scale from 1=“never” to 5=“very often” |
| Low self-control | Low self-control was assessed using 10 items from the Self-Control Scale (adapted by the z-proso team from [5] and recoded, with higher values representing lower levels of self-control (e.g., “I often act on the spur of the moment without stopping to think”). | 0.75 | Mean score: four-point Likert scale from 1=“fully untrue” to 4=“fully true” |
| Delinquency | Delinquency was assessed with a total of 8 self-reported delinquent acts that participants could have engaged in during the past year (e.g., “stolen something at school”). Participants indicated whether they engaged in these behaviors during the past year (yes/no). The items were added up to create a variety score. | -- | Variety/sum score (possible range: 0 to 8) |
| Any substance use | Any substance use in the past year was reported as part of the delinquency scale and coded as 1 if participants reported using alcohol, tobacco, or other substances during the past year. | -- | Binary variable (0= no substance use in past year; 1= use of alcohol, tobacco, or other substances during the past year) |
| Bullying perpetration | Bullying perpetration in the past 12 months was assessed with 4 items assessing physical aggression, teasing, and exclusion [6,7]. | 0.75 | Mean score: six-point Likert scale from 1=“never” to 6=“(almost) every day” |
| **Behavioral protective factors: stress coping and emotional well-being** | | | |
| Low aggressive conflict coping | Aggressive conflict coping was measured with 4 items rated by focal participants. Participants were asked how often, in case of a dispute with others, they engaged in activities such as threatening the other person with punches. Aggressive conflict coping was reverse coded so that higher values reflect low aggressive coping skills. | 0.69 | Mean score: five-point Likert scale from 1=“never” to 5=“very often” |
| Competent conflict coping | Competent conflict coping was measured with 4 items rated by focal participants. Participants were asked how often, in case of a dispute with others, they engaged in activities such as trying to put themselves in the position of the other person to try and understand him or her. | 0.65 | Mean score: five-point Likert scale from 1=“never” to 5=“very often” |
| Emotional well-being | Emotional well-being was self-reported by participants as the frequency of internalizing symptoms on the SBQ [3,4] with 8 items in the past 30 days (e.g., “I couldn’t enjoy anything”). The mean score was reverse coded, with higher scores indicating more emotional well-being. | 0.79 | Mean score: five-point Likert scale from 1=“never” to 5=“very often” |
| **Interpersonal risk factors: experiences of aggression and conflict** | | | |
| Harsh parenting | Participants reported exposure to harsh parenting on 5 items from the Alabama Parenting Questionnaire (APQ; [8] that asked about the frequency of parental corporal punishment (e.g., “When you misbehave or are disobedient, how often do your parents slap you”?), and other forms of harsh parenting (e.g., more severe punishment for no reason; verbal aggression). | 0.66 | Mean score: four-point Likert scale from 1=“never” to 4=“always” |
| Serious victimization | Serious victimization was self-reported on 3 items asking whether they experienced a robbery, assault without a weapon, or assault with a weapon in the past year (yes/no). | -- | Binary variable (0= no experience of serious victimization; 1 = at least one experience of serious victimization) |
| Bullying victimization | Bullying victimization was assessed with 4 items analogous to bullying perpetration with 4 items of the Zurich Bullying Scale [6,7]. | 0.72 | Mean score: six-point Likert scale from 1=“never” to 6=“(almost) every day” |
| Parental relationship quality | Parental relationship quality was rated by the participating parent when children were 8 years old on the Dyadic Adjustment Scale-7 [9,10]. This scale consists of 7 items that assess dyadic consensus (3 items), dyadic cohesion (3 items), and dyadic satisfaction (1 item). A mean of the three subscales was created and then reversed so higher scores indicated poorer parental relationship quality. Only participating parents who were living with a partner were asked about parental relationship quality. | 0.62 | For dyadic consensus and cohesion:  six-point Likert scale from 1=“never” to 6=“always”/”more than once a day”  For dyadic satisfaction:  seven-point Likert scale from 1=“extremely unhappy” to 7=“perfect” |
| Parental disagreement | Parental disagreement was rated when children were 8 years old, with 16 items of the Parent Problem Checklist [11]. The participating parent indicated problem areas between them and their partner or co-parent (if applicable) during the past four weeks. | 0.90 | Mean score: five-point Likert scale from 1=“not a problem” to 5=“a very big problem” |
| School-level exposure to aggression and conflict | School-level problem exposure (e.g., to physical violence, vandalism) was rated by participants’ primary teacher in 4^th^ grade (age 11) on 11 items. | 0.85 | Mean score: five-point Likert scale from 1=“not a problem” to 5=“a very big problem” |
| **Interpersonal protective factors: relationship resources** | | | |
| Parental involvement | Parental involvement was participant-rated on 6 items (e.g., “You play games or do fun things with your parents”) of the APQ [8]. | 0.66 | Mean score: four-point Likert scale from 1=“never” to 4=“often” |
| Teacher bonding | Teacher bonding was assessed with 3 items that asked participants to rate the extent to which certain statements apply to their school experience, including whether they are treated fairly by their teacher. The items were developed by the study team. | 0.78 | Mean score: four-point Likert scale from 1=“fully untrue” to 4=“fully true” |
| Class bonding | Class bonding was assessed with 3 items that asked participants to rate the extent to which certain statements apply to their school experience, including whether they have a good sense of community within the class (*class bonding).* The items were developed by the study team. | 0.77 | Mean score: four-point scale from 1=“fully untrue” to 4=“fully true” |

# **eSupplement 1. Attrition, missing, data, and imputation**

eTable 2 shows group differences on childhood risk and protective factors between those with complete PYPA data and those with partial missing PYPA data.

**eTable 2**. Comparing those with complete PYPA data to those with missing data on PYPA on childhood risk and protective factors

| Variables | Complete PYPA data cases (n=735) | | | | | | | Cases with missing data on PYPA (n=787) | | | | | | | p-value of group difference test^a^ |
| --- | --- | --- | --- | --- | --- | --- | --- | --- | --- | --- | --- | --- | --- | --- | --- |
|  | n | M | SD | n | % | Lower 95% CI | Upper 95%CI | n | M | SD | n | % | Lower 95% CI | Upper 95%CI |  |
| **Sociodemographic risk factors** | | |  |  |  |  |  |  |  |  |  |  |  |  |  |
| Male sex | *--* | *--* | *--* | *355* | 48.3 | 44.6 | 52.0 | *--* | *--* | *--* | 429 | 54.5 | 51.0 | 58.0 | **.015** |
| SES | 724 | 50.03 | 19.86 | *--* | *--* | *--* | *--* | 712 | 41.08 | 17.54 | *--* | *--* | *--* | *--* | **<.001** |
| Migration background (1=both parents born abroad) | *--* | *--* | *--* | 294 | 40.8 | 37.2 | 44.5 |  |  |  | 422 | 58.3 | 54.6 | 61.9 | **<.001** |
| Parental divorce by age 11 | *--* | *--* | *--* | 179 | 24.5 | 21.4 | 27.8 | *--* | *--* | *--* | 113 | 27.6 | 23.3 | 32.2 | .259 |
|  |  |  |  |  |  |  |  |  |  |  |  |  |  |  |  |
| **Behavioral risk** |  |  |  |  |  |  |  |  |  |  |  |  |  |  |  |
| ADHD symptoms | 669 | 1.24 | 0.67 | *--* | *--* | *--* | *--* | 396 | 1.33 | 0.72 | *--* | *--* | *--* | *--* | **.040** |
| Aggression | 735 | 1.53 | 0.40 | *--* | *--* | *--* | *--* | 408 | 1.57 | 0.51 | *--* | *--* | *--* | *--* | .112 |
| Self-control | 735 | 1.93 | 0.44 | *--* | *--* | *--* | *--* | 410 | 1.97 | 0.54 | *--* | *--* | *--* | *--* | .214 |
| Delinquency | 735 | 0.88 | 1.06 | *--* | *--* | *--* | *--* | 412 | 0.79 | 1.06 | *--* | *--* | *--* | *--* | .167 |
| Any substance use | *na* | *na* | *na* | 65 | 8.9 | 6.9 | 11.2 | *na* | *na* | *na* | 31 | 7.5 | 5.2 | 10.5 | .438 |
| Bullying perpetration | 733 | 1.55 | 0.64 | *--* | *--* | *--* | *--* | 411 | 1.52 | 0.70 | *--* | *--* | *--* | *--* | .439 |
|  |  |  |  |  |  |  |  |  |  |  |  |  |  |  |  |
| **Stress coping and emotional well-being** | | | |  |  |  |  |  |  |  |  |  |  |  |  |
| Competent conflict coping | 732 | 3.45 | 0.82 | *--* | *--* | *--* | *--* | 406 | 3.33 | 0.87 | *--* | *--* | *--* | *--* | **.030** |
| Low aggressive conflict coping | 733 | 4.55 | 0.49 | *--* | *--* | *--* | *--* | 408 | 4.50 | 0.63 | *--* | *--* | *--* | *--* | .179 |
| Emotional well-being | 727 | 2.94 | 0.64 | *--* | *--* | *--* | *--* | 409 | 3.00 | 0.70 | *--* | *--* | *--* | *--* | .185 |
|  |  |  |  |  |  |  |  |  |  |  |  |  |  |  |  |
| **Aggression and conflict and exposure** | | | |  |  |  |  |  |  |  |  |  |  |  |  |
| Harsh parenting | 735 | 1.44 | 0.42 | *--* | *--* | *--* | *--* | 411 | 1.46 | 0.45 | *--* | *--* | *--* | *--* | .638 |
| Poor parental relationship quality^a^ | 563 | 2.49 | 0.67 | *--* | *--* | *--* | *--* | 388 | 2.45 | 0.73 | *--* | *--* | *--* | *--* | .427 |
| Parental disagreement^b^ | 605 | 1.53 | 0.46 | *--* | *--* | *--* | *--* | 409 | 1.46 | 0.51 | *--* | *--* | *--* | *--* | **.035** |
| Bullying victimization | 734 | 1.83 | 0.81 | *--* | *--* | *--* | *--* | 411 | 1.69 | 0.75 | *--* | *--* | *--* | *--* | **.005** |
| Serious victimization | *--* | *--* | *--* | 220 | 29.9 | 26.6 | 33.4 | *--* | *--* | *--* | 121 | 29.5 | 25.1 | 34.2 | .882 |
| School problems | 698 | 1.91 | 0.44 | *--* | *--* | *--* | *--* | 517 | 1.94 | 0.45 | *--* | *--* | *--* | *--* | .181 |
|  |  |  |  |  |  |  |  |  |  |  |  |  |  |  |  |
| **Relationship resources** |  |  |  |  |  |  |  |  |  |  |  |  |  |  |  |
| Parental involvement | 735 | 3.36 | 0.47 | *--* | *--* | *--* | *--* | 412 | 3.32 | 0.45 | *--* | *--* | *--* | *--* | .141 |
| Teacher bonding | 731 | 3.47 | 0.57 | *--* | *--* | *--* | *--* | 409 | 3.47 | 0.62 | *--* | *--* | *--* | *--* | .933 |
| Class bonding | 732 | 3.30 | 0.62 | *--* | *--* | *--* | *--* | 409 | 3.40 | 0.57 | *--* | *--* | *--* | *--* | **.005** |

^a^ independent t-test for continuous variables and chi-square test for binary indicators

**Missing Data**

eTable 3 shows the amount of missing data for each analysis variable with respect to the analytic sample of N=1522.

**eTable 3.** Missing data for each variable included in the analysis.

| Variable | Valid n | n/1522 missing | % missing |
| --- | --- | --- | --- |
| PYPA age 11 | 1136 | 386 | 25.4% |
| PYPA age 13 | 1355 | 167 | 11.0% |
| PYPA age 15 | 1438 | 84 | 5.5% |
| PYPA age 17 | 1302 | 220 | 14.5% |
| PYPA age 20 | 1178 | 344 | 22.6% |
| PYPA age 24 | 1159 | 363 | 23.9% |
| **Control variables and sociodemographic risk factors** | | | |
| Intervention: PATHS | 1522 | -- | -- |
| Intervention: PATHS & Triple P | 1522 | -- | -- |
| Intervention: Triple P | 1522 | -- | -- |
| Male sex | 1522 | -- | -- |
| SES | 1436 | 86 | 5.7% |
| Migration background | 1445 | 77 | 5.1% |
| Parental separation by age 11 | 1140 | 382 | 25.1% |
|  |  |  |  |
| **Behavioral risk factors** |  |  |  |
| ADHD symptoms | 1065 | 457 | 30.0% |
| General aggression | 1143 | 379 | 24.9% |
| Low self-control | 1145 | 377 | 24.8% |
| Delinquency | 1147 | 375 | 24.6% |
| Any substance use | 1144 | 378 | 24.8% |
| Bullying perpetration | 1144 | 378 | 24.8% |
|  | |  |  |
| **Aggression and conflict exposure** | |  |  |
| Harsh parenting | 1146 | 376 | 24.7% |
| Poor parental relationship quality^a^ | 951 | 571 | 37.5% |
| Parental disagreement^b^ | 1014 | 508 | 33.4% |
| Bullying victimization | 1145 | 377 | 24.8% |
| Serious victimization | 1145 | 377 | 24.8% |
| School problems | 1215 | 307 | 20.2% |
|  |  |  |  |
| **Stress coping and emotional well-being** | |  |  |
| Competent conflict coping | 1138 | 384 | 25.2% |
| Low aggressive conflict coping | 1141 | 381 | 25.0% |
| Emotional well-being | 1136 | 386 | 25.4% |
|  |  |  |  |
| **Relationship resources** |  |  |  |
| Parental involvement | 1147 | 375 | 24.6% |
| Teacher bonding | 1140 | 382 | 25.1% |
| Class bonding | 1141 | 381 | 25.0% |

**Imputation Model**

We performed multiple imputation stratified by sex (Bayesian method as implemented in Mplus) to address missing data. Initially, we ran models with m=20 imputed data sets. Upon inspecting the fraction of missing information, we increased the number of imputed data sets to m=75, to correspond to guidelines by White et al. (2011) stating that m should equal at least 100 times the fraction of missing information. Data was imputed for the variables PYPA at age 11, PYPA at age 13, PYPA at age 15, PYPA at age 17, PYPA at age 20, PYPA at age 24, SES, migration background, parental separation/divorce, ADHD, general aggression, self-control, delinquency, early substance use, bullying perpetration, harsh parenting, bullying victimization, serious victimization, school problems, competent conflict coping, low aggressive conflict coping, parental involvement, teacher bonding and class bonding. Intervention group was included to inform the imputation of these variables but was not missing. Poor parental relationship quality and parental disagreement were also included to inform the imputation of the other variables, but they were not imputed as not to impute data for parents not living with a partner or co-parenting, respectively. Fraction of Missing Information for imputed risk and protective factors are presented in eTable 4 for models 1 and 2.

**eTable 4. Fraction of Missing Information of focal predictors in models 1 and 2.**

|  | Fraction of Missing Information | |
| --- | --- | --- |
|  | Model 1 | Model 2 |
| Male sex | .10 | .14 |
| SES | .12 | .12 |
| Migration background | .09 | .10 |
| Parental separation by age 11 | .31 | .32 |
|  |  |  |
| **Behavioral risk factors** |  |  |
| ADHD symptoms | .32 | .32 |
| General aggression | .29 | -- |
| Low self-control | .32 | .30 |
| Delinquency | .27 | .25 |
| Any substance use | .32 | .32 |
| Bullying perpetration | .28 | .26 |
| PYPA 11 | .27 | .28 |
|  |  |  |
| **Aggression and conflict exposure** |  |  |
| Harsh parenting | .30 | .30 |
| Bullying victimization | .26 | .28 |
| Serious victimization | .27 | .30 |
| School problems | .29 | .29 |
|  |  |  |
| **Stress coping and emotional well-being** |  |  |
| Competent conflict coping | .29 | .29 |
| Low aggressive conflict coping | .30 | .26 |
| Emotional well-being | .32 | .32 |
|  |  |  |
| **Relationship resources** |  |  |
| Parental involvement | .28 | .27 |
| Teacher bonding | .31 | .33 |
| Class bonding | .29 | .29 |

# **eTable 5. Descriptive statistics for those who ever engaged in PYPA and those who did not engage in PYPA.**

| Variables | No PYPA group (N= 1027) | | | | | | | PYPA group (N= 495) | | | | | | |
| --- | --- | --- | --- | --- | --- | --- | --- | --- | --- | --- | --- | --- | --- | --- |
|  | n | M | SD | n | % | Lower 95% CI | Upper 95%CI | n | M | SD | n | % | Lower 95% CI | Upper 95%CI |
| **PYPA** |  |  |  |  |  |  |  |  |  |  |  |  |  |  |
| Age 11 | *--* | *--* | *--* | *--* | *--* | *--* | -- | *--* | *--* | *--* | 145 | 38.4 | 33.4 | 43.5 |
| Age 13 | *--* | *--* | *--* | *--* | *--* | *--* | -- | *--* | *--* | *--* | 208 | 46.1 | 41.4 | 50.8 |
| Age 15 | *--* | *--* | *--* | *--* | *--* | *--* | -- | *--* | *--* | *--* | 201 | 42.4 | 37.9 | 47.0 |
| Age 17 | *--* | *--* | *--* | *--* | *--* | *--* | -- | *--* | *--* | *--* | 137 | 31.6 | 27.2 | 36.2 |
| Age 20 | *--* | *--* | *--* | *--* | *--* | *--* | -- | *--* | *--* | *--* | 84 | 21.1 | 17.2 | 25.4 |
| Age 24 | *--* | *--* | *--* | *--* | *--* | *--* | -- | *--* | *--* | *--* | 56 | 14.7 | 11.3 | 18.7 |
|  |  |  |  |  |  |  |  |  |  |  |  |  |  |  |
| **Intervention group** | | |  |  |  |  |  |  |  |  |  |  |  |  |
| Paths | *--* | *--* | *--* | 275 | 26.8 | 24.1 | 29.6 | *--* | *--* | *--* | 137 | 27.7 | 23.8 | 31.8 |
| Triple P | *--* | *--* | *--* | 266 | 25.9 | 23.2 | 28.7 | *--* | *--* | *--* | 111 | 22.4 | 18.8 | 26.4 |
| Paths&Triple P | *--* | *--* | *--* | 249 | 24.2 | 21.7 | 27.0 | *--* | *--* | *--* | 104 | 21.0 | 17.5 | 24.9 |
| Control | *--* | *--* | *--* | 237 | 23.1 | 20.5 | 25.8 | *--* | *--* | *--* | 143 | 28.9 | 24.9 | 33.1 |
|  |  |  |  |  |  |  |  |  |  |  |  |  |  |  |
| **Sociodemographic risk factors** | | |  |  |  |  |  |  |  |  |  |  |  |  |
| Male sex | *--* | *--* | *--* | 505 | 49.2 | 46.1 | 52.3 | *--* | *--* | *--* | 279 | 56.4 | 51.9 | 60.8 |
| SES | 971 | 45.41 | 19.28 | *--* | *--* | *--* | -- | 465 | 45.98 | 19.25 | *--* | *--* | *--* | -- |
| Migration background (1=both parents born abroad) | *--* | *--* | *--* | 487 | 50.4 | 47.2 | 53.6 | *--* | *--* | *--* | 229 | 47.8 | 43.3 | 52.4 |
| Parental divorce by age 11 | *--* | *--* | *--* | 190 | 25.0 | 21.9 | 28.2 | *--* | *--* | *--* | 102 | 26.9 | 22.5 | 31.7 |
|  |  |  |  |  |  |  |  |  |  |  |  |  |  |  |
| **Behavioral risk** |  |  |  |  |  |  |  |  |  |  |  |  |  |  |
| ADHD symptoms | 716 | 1.21 | 0.66 | *--* | *--* | *--* | -- | 349 | 1.41 | 0.73 | *--* | *--* | *--* | -- |
| Aggression | 764 | 1.47 | 0.39 | *--* | *--* | *--* | -- | 379 | 1.70 | 0.51 | *--* | *--* | *--* | -- |
| Self-control | 764 | 1.89 | 0.46 | *--* | *--* | *--* | -- | 381 | 2.06 | 0.51 | *--* | *--* | *--* | -- |
| Delinquency | 765 | 0.77 | 1.00 | *--* | *--* | *--* | -- | 382 | 1.02 | 1.16 | *--* | *--* | *--* | -- |
| Any substance use | *--* | *--* | *--* | 53 | 6.9 | 5.2 | 9.0 | *--* | *--* | *--* | 43 | 11.3 | 8.3 | 14.9 |
| Bullying perpetration | 764 | 1.51 | 0.63 | *--* | *--* | *--* | -- | 380 | 1.61 | 0.71 | *--* | *--* | *--* | -- |
|  |  |  |  |  |  |  |  |  |  |  |  |  |  |  |
| **Stress coping and emotional well-being** | | |  |  |  |  |  |  |  |  |  |  |  |  |
| Competent conflict coping | 760 | 3.49 | 0.84 | *--* | *--* | *--* | -- | 378 | 3.24 | 0.81 | *--* | *--* | *--* | -- |
| Low aggressive conflict coping | 763 | 4.59 | 0.52 | *--* | *--* | *--* | -- | 378 | 4.43 | 0.58 | *--* | *--* | *--* | -- |
| Emotional well-being | 758 | 3.00 | 0.66 | *--* | *--* | *--* | -- | 378 | 2.89 | 0.68 | *--* | *--* | *--* | -- |
|  |  |  |  |  |  |  |  |  |  |  |  |  |  |  |
| **Aggression and conflict and exposure** | | | |  |  |  |  |  |  |  |  |  |  |  |
| Harsh parenting | 765 | 1.39 | 0.37 | *--* | *--* | *--* | -- | 381 | 1.57 | 0.50 | *--* | *--* | *--* | -- |
| Poor parental relationship quality^a^ | 642 | 2.43 | 0.69 | *--* | *--* | *--* | -- | 309 | 2.57 | 0.70 | *--* | *--* | *--* | -- |
| Parental disagreement^b^ | 686 | 1.47 | 0.49 | *--* | *--* | *--* | -- | 328 | 1.55 | 0.46 | *--* | *--* | *--* | -- |
| Bullying victimization | 765 | 1.74 | 0.78 | *--* | *--* | *--* | -- | 380 | 1.85 | 0.80 | *--* | *--* | *--* | -- |
| Serious victimization | *--* | *--* | *--* | 194 | 25.4 | 22.3 | 28.6 | *--* | *--* | *--* | 147 | 38.6 | 33.7 | 43.7 |
| School problems | 812 | 1.90 | 0.44 | *--* | *--* | *--* | -- | 403 | 1.96 | 0.44 | *--* | *--* | *--* | -- |
|  |  |  |  |  |  |  |  |  |  |  |  |  |  |  |
| **Relationship resources** | | |  |  |  |  |  |  |  |  |  |  |  |  |
| Parental involvement | 765 | 3.38 | 0.45 | *--* | *--* | *--* | -- | 382 | 3.26 | 0.49 | *--* | *--* | *--* | -- |
| Teacher bonding | 762 | 3.51 | 0.57 | *--* | *--* | *--* | -- | 378 | 3.40 | 0.62 | *--* | *--* | *--* | -- |
| Class bonding | 762 | 3.38 | 0.58 | *--* | *--* | *--* | -- | 379 | 3.25 | 0.64 | *--* | *--* | *--* | -- |

^a^ N=951 because not all parents were living with a partner.

^b^ N=1014 because not all parents were living with a partner or co-parenting.

*Note.* PYPA=physical youth-to-parent aggression.

# **eTable 6. Prevalences of PYPA in the overall sample, and by sex, with confidence intervals**

|  | Overall | | | | Males | | | | Females | | | |
| --- | --- | --- | --- | --- | --- | --- | --- | --- | --- | --- | --- | --- |
|  | n/N | % | Lower 95% CI | Upper 95%CI | n/N | % | Lower 95% CI | Upper 95%CI | n/N | % | Lower 95% CI | Upper 95%CI |
|  |  |  |  |  |  |  |  |  |  |  |  |  |
| Age 11 | 145/1136 | 12.8 | 10.9 | 14.8 | 86/578 | 14.9 | 12.1 | 18.0 | 59/558 | 10.6 | 8.1 | 13.4 |
| Age 13 | 208/1355 | 15.4 | 13.5 | 17.4 | 120/696 | 17.2 | 14.5 | 20.3 | 88/659 | 13.4 | 10.8 | 16.2 |
| Age 15 | 201/1438 | 14.0 | 12.2 | 15.9 | 109/743 | 14.7 | 12.2 | 17.4 | 92/695 | 13.2 | 10.8 | 16.0 |
| Age 17 | 137/1302 | 10.5 | 8.9 | 12.3 | 70/657 | 10.7 | 8.4 | 13.3 | 67/645 | 10.4 | 8.1 | 13.0 |
| Age 20 | 84/1178 | 7.1 | 5.7 | 8.8 | 51/580 | 8.8 | 6.6 | 11.4 | 33/598 | 5.5 | 3.8 | 7.7 |
| Age 24 | 56/1159 | 4.8 | 3.7 | 6.2 | 34/572 | 5.9 | 4.2 | 8.2 | 22/587 | 3.7 | 2.4 | 5.6 |
| Cumulative | 495/1522 | 32.5 | 30.2 | 34.9 | 279/784 | 35.6 | 32,2 | 39.1 | 216/738 | 29.3 | 26.0 | 32.7 |

# **eTable 7. Sensitivity analysis adjusting for PYPA at age 11, intervention group, and sociodemographics**

| Predictors | Adjusted for sex, SES, migration background, treatment group, and PYPA at age 11 | | | p-value: protective factor*YPA 11 interaction |
| --- | --- | --- | --- | --- |
|  | OR | L95%CI | U95%CI |  |
| **Covariates and sociodemographic risk factors** | | |  |  |
| Intervention: PATHS | 0.75 | 0.54 | 1.04 |  |
| Intervention: PATHS & Triple P | 0.73 | 0.52 | 1.03 |  |
| Intervention: Triple P | **0.57** | 0.40 | 0.81 |  |
| Male sex | 1.18 | 0.93 | 1.50 |  |
| SES | 0.91 | 0.79 | 1.04 |  |
| Migration background (1=both parents born abroad) | 1.17 | 0.89 | 1.53 |  |
| Parental divorce by age 11 | 1.04 | 0.75 | 1.44 |  |
|  |  |  |  |  |
| **Behavioral risk** |  |  |  |  |
| ADHD symptoms | **1.25** | 1.09 | 1.45 |  |
| Self-control | 1.14 | 0.99 | 1.32 |  |
| Delinquency | 1.14 | 0.99 | 1.30 |  |
| Any substance use | **1.63** | 1.02 | 2.60 |  |
| Bullying perpetration | 1.01 | 0.89 | 1.16 |  |
|  |  |  |  |  |
| **Stress coping and emotional well-being** | | |  |  |
| Competent conflict coping | **0.83** | 0.72 | 0.95 | .168 |
| Low aggressive conflict coping | 0.88 | 0.77 | 1.01 | .446 |
| Emotional well-being | 0.97 | 0.84 | 1.12 | .460 |
|  |  |  |  |  |
| **Aggression and conflict and exposure** | | |  |  |
| Harsh parenting | **1.24** | 1.09 | 1.42 |  |
| Poor parental relationship quality^a^ | 1.13 | 0.97 | 1.32 |  |
| Parental disagreement^b^ | **1.20** | 1.04 | 1.38 |  |
| Bullying victimization | 1.09 | 0.95 | 1.24 |  |
| Serious victimization | **1.23** | 1.08 | 1.41 |  |
| School problems | 1.06 | 0.92 | 1.21 |  |
|  |  |  |  |  |
| **Relationship resources** | |  |  |  |
| Parental involvement | **0.86** | 0.75 | 0.99 | .991 |
| Teacher bonding | 0.91 | 0.80 | 1.05 | .790 |
| Class bonding | 0.92 | 0.80 | 1.05 | .448 |

^a^ N=951 because not all parents were living with a partner.

^b^ N=1014 because not all parents were living with a partner or co-parenting.

*Note.* SES= socioeconomic status. ADHD= attention-deficit hyperactivity disorder. PYPA= physical youth-to-parent aggression.

| Predictors | Adjusted for sex, SES, migration background, treatment group, and PYPA at age 11 | | |
| --- | --- | --- | --- |
|  | OR | L95%CI | U95%CI |
| **Sociodemographic risk factors** | |  |  |
| Intervention: PATHS | **0.71** | 0.51 | 0.99 |
| Intervention: PATHS & Triple P | 0.71 | 0.51 | 1.01 |
| Intervention: Triple P | **0.56** | 0.39 | 0.79 |
| Male sex | 1.01 | 0.78 | 1.30 |
| SES | 0.92 | 0.80 | 1.05 |
| Migration background (1=both parents born abroad) | 1.15 | 0.87 | 1.51 |
| Parental divorce by age 11 | 0.99 | 0.71 | 1.38 |
|  |  |  |  |
| **Behavioral risk** |  |  |  |
| ADHD symptoms | **1.22** | 1.06 | 1.42 |
| Self-control | 0.97 | 0.83 | 1.15 |
| Delinquency | 1.02 | 0.88 | 1.17 |
| Any substance use | 1.37 | 0.84 | 2.24 |
| Bullying perpetration | **0.83** | 0.70 | 0.98 |
|  |  |  |  |
| **Stress coping and emotional well-being** | | |  |
| Competent conflict coping | 0.87 | 0.76 | 1.01 |
| Low aggressive conflict coping | 1.12 | 0.93 | 1.33 |
| Emotional well-being | 1.06 | 0.91 | 1.24 |
|  |  |  |  |
| **Aggression and conflict exposure** | |  |  |
| Harsh parenting | 1.15 | 0.99 | 1.32 |
| Poor parental relationship quality^a^ | 1.09 | 0.93 | 1.28 |
| Parental disagreement^b^ | **1.17** | 1.01 | 1.36 |
| Bullying victimization | 1.00 | 0.87 | 1.16 |
| Serious victimization | **1.17** | 1.01 | 1.34 |
| School problems | 1.04 | 0.90 | 1.19 |
|  |  |  |  |
| **Relationship resources** | |  |  |
| Parental involvement | 0.88 | 0.77 | 1.02 |
| Teacher bonding | 1.00 | 0.86 | 1.15 |
| Class bonding | 0.98 | 0.85 | 1.13 |

# **eTable 8. Sensitivity analysis adjusting for PYPA at age 11, general aggression, intervention group, and sociodemographics**

^a^ N=951 because not all parents were living with a partner.

^b^ N=1014 because not all parents were living with a partner or co-parenting.

*Note.* SES= socioeconomic status. ADHD= attention-deficit hyperactivity disorder. PYPA= physical youth-to-parent aggression.

**eTable 9. Results from complete case analysis.**

| Risk factors | Model 1 | | | | Model 2 | | |
| --- | --- | --- | --- | --- | --- | --- | --- |
|  | OR | L95%CI | U95%CI | Sex interaction (p) | OR | L95%CI | U95%CI |
| **Control variables and sociodemographic risk factors** | | | | |  |  |  |
| Intervention: PATHS | **0.67** | 0.45 | 0.99 | -- | **0.60** | 0.38 | 0.93 |
| Intervention: PATHS & Triple P | 0.70 | 0.47 | 1.06 | -- | 0.64 | 0.40 | 1.02 |
| Intervention: Triple P | **0.47** | 0.31 | 0.72 | -- | **0.49** | 0.31 | 0.78 |
| Male sex | 1.07 | 0.80 | 1.44 | -- | 0.90 | 0.64 | 1.27 |
| SES | 0.96 | 0.82 | 1.13 | .742 | 0.96 | 0.81 | 1.15 |
| Migration background | 1.18 | 0.86 | 1.64 | .926 | 1.22 | 0.85 | 1.74 |
| Parental separation by age 11 | 1.22 | 0.82 | 1.80 | .446 | 1.19 | 0.81 | 1.76 |
|  |  |  |  |  |  |  |  |
| **Behavioral risk factors** | |  |  |  |  |  |  |
| ADHD symptoms | **1.37** | 1.15 | 1.63 | .906 | **1.33** | 1.11 | 1.59 |
| General aggression | **1.44** | 1.20 | 1.74 | .309 | **1.44** | 1.20 | 1.74 |
| Low self-control | **1.22** | 1.01 | 1.47 | .093 | 1.03 | 0.84 | 1.28 |
| Delinquency | **1.21** | 1.03 | 1.42 | .808 | 1.09 | 0.91 | 1.30 |
| Any substance use | 1.65 | 0.95 | 2.88 | **.027** | 1.41 | 0.78 | 2.57 |
| Bullying perpetration | 1.06 | 0.88 | 1.27 | .642 | 0.83 | 0.66 | 1.04 |
| PYPA 11 | **5.52** | 3.43 | 8.89 | .168 | **4.87** | 3.00 | 7.91 |
|  |  |  |  |  |  |  |  |
| **Aggression and conflict exposure** | | |  |  |  |  |  |
| Harsh parenting | **1.39** | 1.18 | 1.63 | **.040** | **1.29** | 1.09 | 1.52 |
| Poor parental relationship quality^a^ | **1.21** | 1.00 | 1.45 | .127 | 1.14 | 0.93 | 1.39 |
| Parental disagreement^b^ | 1.16 | 0.98 | 1.36 | .205 | 1.14 | 0.95 | 1.38 |
| Bullying victimization | 1.13 | 0.97 | 1.30 | .656 | 1.02 | 0.86 | 1.21 |
| Serious victimization | **1.36** | 1.16 | 1.60 | .755 | **1.27** | 1.07 | 1.50 |
| School problems | 1.06 | 0.90 | 1.24 | .822 | 1.02 | 0.86 | 1.22 |
|  |  |  |  |  |  |  |  |
| **Stress coping and emotional well-being** | | |  |  |  |  |  |
| Competent conflict coping | **0.79** | 0.67 | 0.93 | .621 | 0.85 | 0.71 | 1.01 |
| Low aggressive conflict coping | 0.88 | 0.73 | 1.05 | .785 | 1.18 | 0.93 | 1.49 |
| Emotional well-being | 0.88 | 0.74 | 1.04 | .297 | 0.97 | 0.81 | 1.16 |
|  |  |  |  |  |  |  |  |
| **Relationship resources** | |  |  |  |  |  |  |
| Parental involvement | **0.81** | 0.69 | 0.96 | .083 | **0.80** | 0.68 | 0.95 |
| Teacher bonding | 0.95 | 0.80 | 1.13 | .348 | 1.04 | 0.87 | 1.25 |
| Class bonding | **0.84** | 0.72 | 0.98 | .360 | 0.90 | 0.76 | 1.06 |

*Note.* SES= socioeconomic status. ADHD= attention-deficit hyperactivity disorder. PYPA= physical youth-to-parent aggression. Range of n for Model 1 n= 607-882 and for Model 2 n=556-714.

# **References**

1. Ribeaud D, Murray A, Shanahan L, Shanahan MJ, Eisner M. Cohort Profile: The Zurich Project on the Social Development from Childhood to Adulthood (z-proso). J Dev Life-Course Criminol. 2022;8:151–71. https://doi.org/10.1007/s40865-022-00195-x.

2. Ganzeboom HBG, De Graaf PM, Treiman DJ. A standard international socio-economic index of occupational status. Soc Sci Res. 1992;21:1–56. https://doi.org/10.1016/0049-089X(92)90017-B.

3. Tremblay RE, Loeber R, Gagnon C, Charlebois P, Larivée S, LeBlanc M. Disruptive boys with stable and unstable high fighting behavior patterns during junior elementary school. J Abnorm Child Psychol. 1991;19:285–300. https://doi.org/10.1007/BF00911232.

4. Murray AL, Obsuth I, Eisner M, Ribeaud D. Evaluating Longitudinal Invariance in Dimensions of Mental Health Across Adolescence: An Analysis of the Social Behavior Questionnaire. Assessment. 2019;26:1234–45. https://doi.org/10.1177/1073191117721741.

5. Grasmick HG, Tittle CR, Bursik RJ, Arneklev BJ. Testing the Core Empirical Implications of Gottfredson and Hirschi’s General Theory of Crime. J Res Crime Delinquency. 1993;30:5–29. https://doi.org/10.1177/0022427893030001002.

6. Murray AL, Eisner M, Ribeaud D, Kaiser D, McKenzie K, Murray G. Validation of a Brief Self-Report Measure of Adolescent Bullying Perpetration and Victimization. Assessment. 2021;28:128–40. https://doi.org/10.1177/1073191119858406.

7. Olweus, D. Bullying at school: What we know and what we can do. Malden, MA: Blackwell Publishing; 1993.

8. Shelton KK, Frick PJ, Wootton J. Assessment of parenting practices in families of elementary school age children. J Clin Child Psychol. 1996;25:317–29.

9. Hunsley J, Best M, Lefebvre M, Vito D. The seven-item short form of the Dyadic Adjustment Scale: Further evidence for construct validity. Am J Fam Ther. 2001;29:325–35.

10. Spanier GB. Measuring dyadic adjustment: New scales for assessing the quality of marriage and similar dyads. J Marriage Fam. 1976;15–28.

11. Dadds MR, Powell MB. The relationship of interparental conflict and global marital adjustment to aggression, anxiety, and immaturity in aggressive and nonclinic children. J Abnorm Child Psychol. 1991;19:553–67. https://doi.org/10.1007/BF00925820.
